# Overexpression of Cystine/Glutamate Antiporter xCT Correlates with Nutrient Flexibility and ZEB1 Expression in Highly Clonogenic Glioblastoma Stem-like Cells (GSCs)

**DOI:** 10.3390/cancers13236001

**Published:** 2021-11-29

**Authors:** Katharina Koch, Rudolf Hartmann, Abigail Kora Suwala, Dayana Herrera Rios, Marcel Alexander Kamp, Michael Sabel, Hans-Jakob Steiger, Dieter Willbold, Amit Sharma, Ulf Dietrich Kahlert, Jarek Maciaczyk

**Affiliations:** 1 Department of Neurosurgery, University Hospital Duesseldorf, 40225 Duesseldorf, Germany; Katharina.Koch@IUF-Duesseldorf.de (K.K.); Dayana.Herrera-Rios@uk-essen.de (D.H.R.); Michael.Sabel@med.uni-duesseldorf.de (M.S.); Hans-Jakob.Steiger@med.uni-duesseldorf.de (H.-J.S.); ulf.kahlert@med.ovgu.de (U.D.K.); 2 IUF—Leibniz Research Institute for Environmental Medicine, 40225 Duesseldorf, Germany; 3 Institute of Biological Information Processing (IBI-7) Structural Biochemistry, Forschungszentrum Juelich, 52425 Juelich, Germany; r.hartmann@fz-juelich.de (R.H.); d.willbold@fz-juelich.de (D.W.); 4 Department of Neurological Surgery, Helen Diller Research Center, University of California San Francisco, San Francisco, CA 94158, USA; abigail.suwala@med.uni-heidelberg.de; 5 Department of Neuropathology, Institute of Pathology, Heidelberg University Hospital, 69120 Heidelberg, Germany; 6 Clinical Cooperation Unit Neuropathology, German Cancer Research Center (DKFZ), German Consortium for Translational Cancer Research (DKTK), 69120 Heidelberg, Germany; 7 Skin Cancer Unit of the Dermatology Department, West German Cancer Center, University Duisburg-Essen, 45147 Essen, Germany; 8 Department of Neurosurgery, Centre of Neuro-Oncology, Jena University Hospital, Friedrich-Schiller-University Jena, 07747 Jena, Germany; MarcelAlexander.Kamp@med.uni-jena.de; 9 Institut für Physikalische Biologie, Heinrich-Heine-University Duesseldorf, 40225 Duesseldorf, Germany; 10 Department of Neurosurgery, University Hospital Bonn, 53127 Bonn, Germany; Amit.Sharma@ukbonn.de; 11 Department of Molecular and Experimental Surgery, Clinic of General, Visceral, Vascular and Transplant Surgery, Faculty of Medicine, Otto-von-Guericke University and University Hospital Magdeburg, 39120 Magdeburg, Germany; 12 Department of Surgical Sciences, University of Otago, Dunedin 9054, New Zealand

**Keywords:** glioblastoma, cancer stem cells, NMR spectroscopy, glutamine, metabolism, xCT, ZEB1, oncometabolites

## Abstract

**Simple Summary:**

Glioblastoma (GBM) is the most aggressive form of glioma (WHO grade IV), and mounting evidence suggests that glioblastoma stem-like cells (GSCs) play an important role in tumor growth and response to therapy. In the current study, we sought to understand the metabolic dependencies of GSCs using high-resolution proton magnetic resonance spectroscopy (^1^H-NMR). In a defined experimental setting, we stratified in vitro GSC models into two subtypes (Gln/Glu^High^, Gln/Glu^Low^) and used diverse molecular approaches to perform comprehensive analyses in GSC neurosphere cultures and primary GBM samples.

**Abstract:**

Cancer stem-like cells mediate tumor initiation, progression, and therapy resistance; however, their identification and selective eradication remain challenging. Herein, we analyze the metabolic dependencies of glioblastoma stem-like cells (GSCs) with high-resolution proton nuclear magnetic resonance (^1^H-NMR) spectroscopy. We stratify our in vitro GSC models into two subtypes primarily based on their relative amount of glutamine in relationship to glutamate (Gln/Glu). Gln/Glu^High^ GSCs were found to be resistant to glutamine deprivation, whereas Gln/Glu^Low^ GSCs respond with significantly decreased in vitro clonogenicity and impaired cell growth. The starvation resistance appeared to be mediated by an increased expression of the glutamate/cystine antiporter SLC7A11/xCT and efficient cellular clearance of reactive oxygen species (ROS). Moreover, we were able to directly correlate xCT-dependent starvation resistance and high Gln/Glu ratios with in vitro clonogenicity, since targeted differentiation of GSCs with bone morphogenic protein 4 (BMP4) impaired xCT expression, decreased the Gln/Glu ratio, and restored the sensitivity to glutamine starvation. Moreover, significantly reduced levels of the oncometabolites lactate (Lac), phosphocholine (PC), total choline (tCho), myo-inositol (Myo-I), and glycine (Gly) were observed in differentiated GSCs. Furthermore, we found a strong association between high Gln/Glu ratios and increased expression of Zinc finger E-box-binding homeobox 1 (ZEB1) and xCT in primary GBM tumor tissues. Our analyses suggest that the inhibition of xCT represents a potential therapeutic target in glioblastoma; thus, we could further extend its importance in GSC biology and stress responses. We also propose that monitoring of the intracellular Gln/Glu ratio can be used to predict nutrient stress resistance.

## 1. Introduction

Glioblastoma (GBM) is the most common malignant primary brain tumor, with a median overall survival of less than two years. Despite recent advances in treatment strategies for this grade IV glioma, the improvement of recurrence rates and survival has reached a plateau. The high frequency of therapy resistance and tumor relapse in GBM is a major clinical challenge attributable to glioblastoma stem-like cells (GSCs), a cell subpopulation that is highly resistant to standard therapies [1,2]. Though molecular sub-classification has improved our understanding of GBM biology, complete strategies to identify and target GSCs are still lacking [3,4]. The prime reason could well be the diverse and dynamic molecular signatures, metabolic phenotypes, and biological responses that GSCs are presumed to exhibit [5,6,7,8].

Considering that the ability of stem cells to self-renew, proliferate, and differentiate is highly dependent on metabolic, especially glycolytic, flexibility, some attempts have been made to study the metabolic pathways associated with GSCs. For instance, Vlashi et al. reported that GSCs and their progenitor cells were less glycolytic compared to differentiated glioma cells [9]. Similarly, a study revealed that insulin-like growth factor 2 mRNA-binding protein 2 (Imp2) regulates oxidative phosphorylation (OXPHOS) in primary glioblastoma GSC spheres (gliomasphere) [10]. Given that not only glucose but also glutamine (Gln) are critical nutrients for cancer cell growth, the molecular mechanism by which cancer cells adapt to glutamine is an intriguing issue, knowing that glutamine metabolism is deregulated in cancer [11,12,13]. Gln is hydrolytically deamidated to glutamate (Glu) in the first step of glutaminolysis. Glu is then either fueled into the tricarboxylic acid (TCA) cycle, used as a nitrogen-donor during the synthesis of non-essential amino acids, or exported out of the cell by the glutamate/cystine antiporter SLC7A11/xCT, thereby promoting glutathione (GSH) synthesis and the maintenance of redox homeostasis. However, glutamine dependency does not apply to all tumors and the microenvironment can significantly modulate the tumor cells’ response to Gln deprivation. Several studies describe Gln addiction in GBM, showing profound anti-proliferative effects in GBM cultures deprived of extracellular Gln or treated with pharmacological inhibitors of glutaminolysis [14,15,16,17,18,19]. As an example, Wise et al. observed that the viability of the SF188 glioma cell line strictly depends on glutamine catabolism [12]. Interestingly, another study reported that the Gln requirements of GBM extend beyond the metabolite supply to the TCA cycle (e.g., anaplerosis, a process that features the formation of TCA cycle intermediates), and moreover identified two alternative metabolic determinants of Gln sensitivity [20]. Oizil et al. were further able to stratify GSCs into GLN-low and GLN-high GSCs on the basis of their glutamine consumption rate [21]. Furthermore, GSC-interdependent metabolic plasticity is still an emerging area of research.

Herein, we sought to understand the metabolic dependencies of GSCs using high-resolution proton magnetic resonance spectroscopy (^1^H-NMR). In a defined experimental framework, we stratified our in vitro GSC models into two subtypes (Gln/Glu^High^, Gln/Glu^Low^) and used diverse molecular approaches to perform comprehensive analyses in GSC neurosphere cultures and primary GBM samples.

## 2. Materials and Methods

### 2.1. Cell Culture

JHH520 cells were generously provided by G. Riggins (Baltimore, MD, USA), GBM1 by A. Vescovi (Milan, Italy), 407 by M.S. Carro (Freiburg, Germany), and RAV19 by M. Proescholdt (Regensburg, Germany). GSC neurospheres were cultured in DMEM w/o pyruvate (Gibco, Thermo Fisher Scientific, Waltham, MA, USA), 30% Ham’s F12 Nutrient Mix (Gibco), 2% B27 supplement (Gibco), 20 ng/mL human bFGF (Peprotech, Rocky Hill, NJ, USA), 20 ng/mL human EGF (Peprotech), 5 µg/mL Heparin (Sigma, Merck KGaA, Darmstadt, Germany), and 1× Anti-Anti (Gibco). All cells were cultured at 37 °C and 5% CO_2_. GSCs were re-differentiated for 48 h with 50 ng/mL recombinant BMP4 (Gibco, #PHC9534) in neurosphere medium. BMP4 was stored as a stock of 1 mg/mL in 10 mM citric acid (pH 3.0). GSCs were starved for glutamine via cultivation in neurosphere medium containing DMEM w/o L-glutamine (Gibco, #11960044) and medium supplemented with 2.6 mM L-glutamine as a control. Primary GBM tumor samples were frozen in liquid nitrogen until lysates were prepared and metabolite extraction was performed. The study was conducted in accordance with the Declaration of Helsinki, and the protocol was approved by the Ethics Committee of the Medical Faculty of the Heinrich-Heine University (#2019-484-FmB and Study ID #5206). Informed consent for inclusion was given by all subjects before they participated in the study.

### 2.2. Generation of Lentiviral Particles

In order to generate the lentiviral particles for shRNA-mediated knockdown of xCT and the respective control particles, we applied the third-generation lentiviral packaging system, as described previously [22]. In brief, we transfected HEK293T cells with the respective lentiviral expression vector (pLKO.1-shxCT or pLKO.1 TCR empty vector) and three different packaging plasmids (pMDLgpRRE, pRSVREV, and pMD2VSVG), using FuGENE^®^ HD Transfection Reagent (Promega, Fitchburg, WI, USA). Exactly 48 h, 72 h, and 96 h post-transfection, the virus supernatant was collected, concentrated, and stored at −80 °C until use. The short hairpin RNA (shRNA) sequences against xCT were designed with the software Primer3 and cloned into the pLKO.1 TRC vector (Addgene plasmid #10878).

### 2.3. Quantitative Real-Time PCR

For the analysis of mRNA expression after exposure to BMP4, we performed quantitative real-time PCR as described previously [23]. Therefore, we extracted total RNA using the NucleoSpin^®^ RNA Kit (Macherey-Nagel, Dueren, Germany) according to the manufacturer’s instructions. Complementary cDNA single strands were synthesized from two µg total RNA using M-MLV reverse transcriptase (Promega). The quantitative real-time PCR was performed using 10 ng cDNA, 10 pmol/primer, and 1× SYBR Green (BioRad, Hercules, CA, USA, #1725272) in a CFX Connect Thermocycler (BioRad). Using the delta delta C(T) method, we normalized the relative quantifications to the expression of the endogenous housekeeping genes β-actin and β-2-microglobulin using the supplied software of the CFX Connect Real-Time PCR Detection System (Bio-Rad).

### 2.4. Western Blotting

Cell lysates were electrophoretically separated via SDS PAGE and transferred onto nitrocellulose membranes, as described previously [23]. We applied the following primary antibodies and used the indicated dilutions: ZEB1 (1:2000, Sigma, #HPA027524), CD133 (1:250, Miltenyi Biotec, Bergisch Gladbach, Germany, #W6B3C1), c-Myc (1:1000, Thermo Fisher Scientific, #9E10), SOX2 (1:1000, Cell Signaling Technology, Cambridge, UK, #L1D6A2), xCT (1:1000, Thermo Fisher Scientific, #PA1-16893 and 1:1000, Cell Signaling Technologies, #12691), CD44 (1:2000, R&D Systems, Minneapolis, MN, USA, #AF3660), and β-actin (Thermo Fisher Scientific, #MA5-15739 and Cell Signaling Technology, #4970) were incubated overnight at 4 °C in 5% milk powder in Tris-buffered saline + 0.1% Tween-20 (TBST). The secondary antibodies goat-anti-rabbit IRDye800CW (1:10,000, LI-COR, Lincoln, NE, USA, #926-32211), goat-anti-mouse IRDye680RD (1:10,000, LI-COR #926-68070), donkey-anti-goat IRDye800CW (1:10,000, LI-COR, #926-32214), goat anti-rabbit-HRP (1:10,000, Jackson ImmunoResearch, Ely, UK, #111-035-144), and goat anti-mouse-HRP (1:10,000, Jackson ImmunoResearch, #111-035-003) were diluted in 5% milk powder in TBST and incubated for 1 h at room temperature. Chemiluminescent signals were detected on a film-based system using chemiluminescent substrates (Thermo Fisher Scientific, #34,096). Fluorescence-labeled antibodies were detected with a LI-COR Odyssey CLx Imager (LI-COR). Precision Plus Protein™ WesternC™ (BioRad, #1610376) was used as a molecular weight marker. Densitometry was performed with the supplied software from LI-COR or ImageJ software [24]. Protein expression was normalized to β-actin for densitometry analysis. The original Western blots can be seen in the supplementary files (Figures S1–S6).

### 2.5. Dual-Phase Metabolite Extraction

Water-soluble metabolites were extracted as previously described [23,25,26]. In brief, a minimum of 5 × 10^6^ cells were harvested, washed with PBS, and extracted with the dual-phase methanol/chloroform/water (1:1:1, *v/v/v*) method. Subsequently, the cells were washed twice with 5 mL ice-cold 0.9% NaCl, re-suspended in 850 µL ice-cold ddH_2_O, and transferred into pre-chilled glass tubes. Afterwards, 4 mL of ice-cold methanol were added, the tubes were vortexed vigorously and incubated on ice for 15 min. After the addition of 4 mL of ice-cold chloroform, the glass tubes were vortexed and incubated for another 10 min on ice. Finally, 3.15 mL of ice-cold ddH_2_O were added, vortexed, and incubated overnight at 4 °C. After centrifugation for 30 min at 4 °C and 4500 rpm, the upper water-methanol phase was separated and incubated for 10 min with 10 mg Chelex^®^ 100 resin (Sigma) on ice. The samples were filtered through a 70 µm mesh which retained the Chelex and the methanol was evaporated for 1 h at 30 °C in a vacuum concentrator. Finally, the samples were frozen in liquid nitrogen, lyophilized, and stored at −20 °C until the spectroscopy measurements.

### 2.6. NMR Data Acquisition and Processing

The acquisition of spectra was performed as described previously [23]. Prior to the measurements, the lyophilisates were re-suspended in 20 mM phosphate buffer (pH 7.0) containing 10% D_2_O and 3-(Trimethylsilyl) propionic acid (TSP; Lancaster Synthesis, Ward Hill, MA, USA) as an internal standard, as described previously [23]. Briefly, one-dimensional ^1^H-NMR spectra were acquired using a Bruker AVANCE III HD 700 spectrometer (Bruker, Ettlingen, Germany) equipped with a 5 mm HCN TCI cryo-probe operating at 700 MHz (16.4 Tesla). The ^1^H-NMR datasets were obtained using excitation sculpting for water suppressing and the following acquisition parameters: 25 °C sample temperature, 9800 Hz sweep width, 256 transients with 32 K time-domain data points were accumulated with a repetition time of 3.2 s. Mestrenova version 8.0.1–10878 (Mestrelab Research S.L, Santiago de Compostela, Spain) software was used to process and analyze the ^1^H-NMR spectra. Equal concentrations of TSP in each sample were used as an internal standard for normalization. The figures show ^1^H-NMR data from a minimum of three independent experiments presented as mean ± SD and statistical significance was calculated with unpaired Student *t*-tests. Regarding the stratification of the GSC lines in Gln/Glu^High^ and Gln/Glu^Low^ cells, we first assessed the Gln and Glu levels in comparison to the TSP standard. Therefore, we integrated the respective multiplets for Gln (ca. 2.4–2.5 ppm) and Glu (ca. 2.3–2.4 ppm) and normalized them against the integral of the TSP standard. The exact location of the Gln and Glu multiplets within the spectrum of water-soluble metabolites can be seen in [23]. From the obtained values, we calculated the ratio of Gln and Glu, compared it in GSC cell lines, and divided them into Gln/Glu^High^ and Gln/Glu^Low^. To mention, we took the ratio of 1 as a cutoff value, but whether this ratio is applicable to future patient measurements remains to be investigated.

### 2.7. Cell Viability and Apoptosis Assays

Cell viability was assessed as the mitochondrial activity of the cells, as described previously [23]. In brief, triplicates of 10,000 cells per well were plated into a 96-well plate. To assess the effect of glutamine starvation, we plated the cells in neurosphere medium either with or without L-glutamine. To asses if Glu and αKG rescue the starvation phenotype, either 4 mM Glu (Sigma, #G1251-100G) or 4 mM αKG (Sigma, #7589-25G) were added to the neurosphere medium with or without L-glutamine. Every other day, the viable cell mass was assessed using the CellTiter-Blue^®^ Cell Viability Assay (Promega, #G8081) or thiazolyl blue tetrazolium bromide (MTT) (Sigma, #2128-1G) according to the manufacturer’s instructions. For CellTiter-Blue, the fluorescence was measured at 560ex/590em and for MTT absorbance was measured at 570 nm (reference 650 nm) using a Safire 2 multiplate reader (Tecan, Maennedorf, Switzerland) as described previously [23].

### 2.8. Clonogenicity Assays

The clonogenicity of GSCs was assessed with colony-forming assays in semi-solid agarose medium, as described previously [23]. Six-well plates were coated with 1.5 mL of 1% agarose (Gibco, #18300012) in pre-warmed neurosphere medium to generate a solid base-layer. After solidification for 1 h at RT, 2 mL of a single-cell suspension (3500 cells/well) in 0.6% agarose in neurosphere medium were added. The agarose must not be hotter than 37 °C to avoid cell damage. The cell layer was incubated for 1 h at room temperature, forming a semi-solid gel. Afterwards, 2 mL neurosphere medium were added on top, supplying the cells within the gel with nutrients. To test GSC clonogenicity after glutamine starvation, all layers were prepared with neurosphere medium either with or without L-glutamine. To test the effect of BMP4 and NAC on GSC clonogenicity, we either added 50 ng/mL BMP4, an equal volume of citric acid, or 3 mM NAC to the upper medium layer. Twice a week the upper layer was removed and 2 mL neurosphere medium containing the specific treatment were added. After three weeks the top medium layer was removed, replaced by 1 mL of 1 mg/mL 4-Nitro blue tetrazolium chloride (NBT) (Sigma) in PBS, and incubated overnight at 37 °C under standard cell culture conditions. In the case of the starvation experiments in the presence of BMP4, we had to expand the incubation period to 5 weeks to yield enough colonies for analysis. The stained colonies were counted using the Clono Counter software [27].

### 2.9. ROS Assays

The accumulation of ROS under Gln starvation was assessed using 2′,7′-dichlorofluorescin diacetate (DCFDA) (Sigma, #D6883) as described previously [28]. In this study, the cells were incubated for 48 h in neurosphere medium with or without L-glutamine under standard cell culture conditions. Subsequently, 3 × 10^5^ cells per condition were washed with PBS, resuspended in PBS containing 50µM DCFDA, and incubated for 30 min in the incubator at 37 °C, to mediate the uptake of DCFDA by the cells. After incubation, the cells were washed with PBS and triplicates of each 1 × 10^5^ cells in 200 µL PBS were transferred into a black 96-well microplate with clear bottom. DCFDA fluorescence was measured at 493ex/515em using a Safire 2 multiplate reader (Tecan).

### 2.10. Statistical Analyses

**Figure 1:**
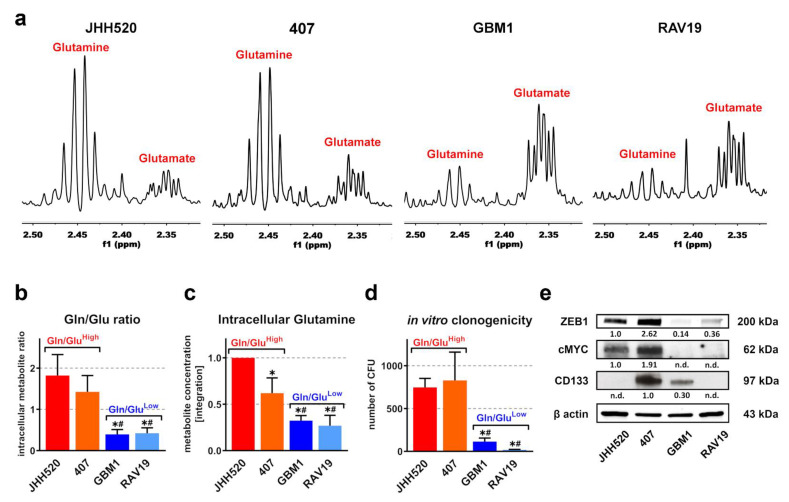
High Gln/Glu ratios predicted for in vitro clonogenicity in glioblastoma stem-like cells (GSCs). (**a**) Expanded regions from ^1^H-NMR spectra showing Gln and Glu multiplets and (**b**) corresponding Gln/Glu ratios of metabolic extracts from untreated JHH520, 407, GBM1, and RAV19 GSC cultures (*n* ≥ 6). (**c**) Relative intracellular Gln concentrations were assessed with ^1^H-NMR spectroscopy (*n* = 3). (**d**) The baseline soft agar colony formation capacity (in vitro clonogenicity) of untreated GSC cultures showed correlation with high Gln/Glu ratios (*n* = 9). (**e**) Immunoblotting of baseline ZEB1, CD133, and cMYC protein showed elevated ZEB1 and cMYC in GSCs with high Gln/Glu ratios (JHH520 and 407) (loading control = β actin). For all assays, the data are presented as mean ± SD. Significance was calculated with a one-way ANOVA, * significantly decreased (*p* < 0.05) compared to JHH520, # significantly decreased (*p* < 0.05) compared to 407. Abbreviations: Gln, glutamine; Glu, glutamate; n.d., not detectable; ppm, parts per million. Supplementary Figure 1 (S1) corresponds to Figure 1e.

All statistics were performed with GraphPad Prism Software Version 8.0.2 (GraphPad Software Inc., San Diego, CA, USA). All results are presented as mean ± SD of a minimum of three independent biological replicates (exact numbers indicated in the figure legends). To calculate statistical significance in an experiment with two conditions (treated vs. untreated) we performed unpaired two-tailed Student’s *t*-tests. When more than two conditions were compared to each other (cell line comparison in Figure 1 and Figure 3), we performed one-way ANOVA and Dunnett’s and Bonferroni’s multiple comparison tests. For all experiments, significance was defined as a *p*-value below 0.05.

## 3. Results

### 3.1. In Vitro Clonogenicity of GSCs Directly Correlates with High Gln/Glu Ratios and Elevated Intracellular Glutamine Concentrations

First, we analyzed the metabolic composition of four GSC neurosphere cultures (JHH520, 407, GBM1, and RAV19) using ^1^H-NMR spectroscopy (Figure 1). The extended regions of the ^1^H-NMR spectra showed distinct Gln and Glu multiplets (Figure 1a). In addition, JHH520 and 407 cells showed higher Gln/Glu ratios and intracellular Gln compared to GBM1 and RAV19 cells (Figure 1b,c). Higher Gln/Glu ratios further correlated with increased in vitro clonogenicity, which is considered the major stemness phenotype of GSCs (Figure 1d). Additionally, ZEB1 protein expression was found to be elevated in clonogenic Gln/Glu^High^ cells (Figure 1e). ZEB1 is a transcription factor that drives epithelial-mesenchymal transition (EMT), and is involved in increased invasiveness and GSC enrichment [29]. Moreover, we observed increased expression of the oncogene c-Myc (a regulator of glucose and glutamine metabolism) in Gln/Glu^High^ GSCs. Notably, the in vitro clonogenicity of our GSC cultures did not correlate with the expression of another putative GSC marker prominin/CD133. Overall, we identified two GSC subsets: highly clonogenic Gln/Glu^High^ cells (JHH520 and 407), which showed high amounts of ZEB1 and c-Myc, and low clonogenic Gln/Glu^Low^ cells (GBM1), that exhibited significantly less ZEB1 and c-Myc protein.

### 3.2. Gln/Glu^High^ GSCs Are Apparently Self-Sustaining for Glutamine

**Figure 2:**
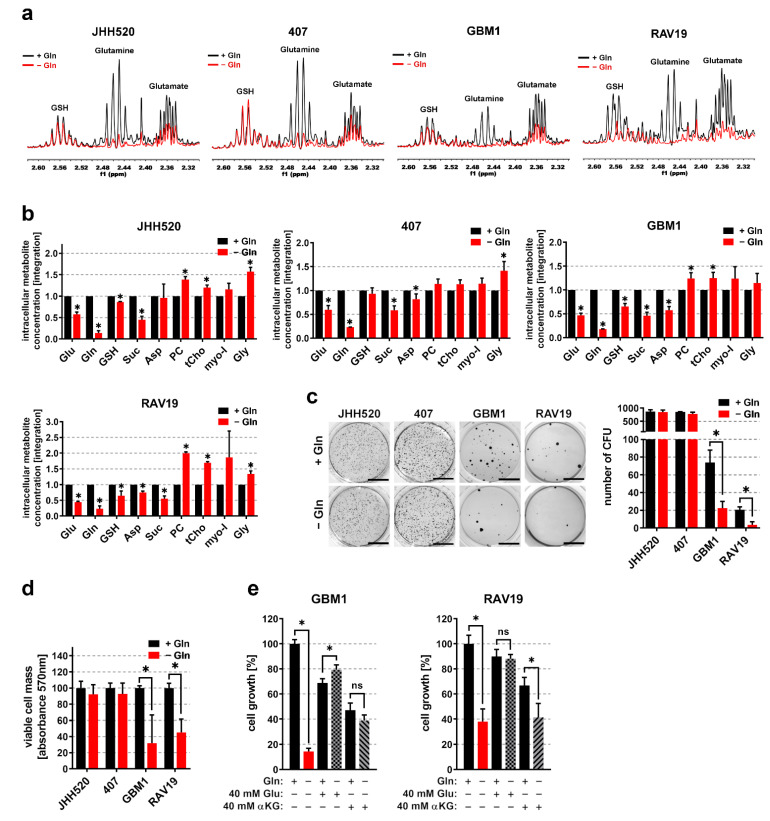
Starvation for L-glutamine targets barely clonogenic GSCs with low Gln/Glu ratios. Expanded regions from ^1^H-NMR spectra showing GSH, Gln, and Glu multiplets (**a**) and relative metabolite concentrations (**b**) from metabolic extracts of GSC neurospheres cultured in the presence (black) or absence (red) of L-Gln (*n* = 3). Gln starvation significantly reduced the in vitro clonogenicity (**c**) and cell growth (**d**) of GSCs with low Gln/Glu ratios (GBM1 and RAV19) (*n* = 3). For in vitro clonogenicity experiments, representative pictures of NBT-stained colonies and quantifications of three soft agar colony formation assays are shown. Scale bar equals 1 cm. (**e**) Cell growth of sensitive GBM1 and RAV19 cells starved for 4 days for L-Gln in the presence of either 4 mM Glu, 4 mM αKG, or medium only (*n* ≥ 3). For all assays, the data are presented as mean ± SD. Significance was calculated with Student’s *t*-test, * *p* < 0.05. Abbreviations: αKG, alpha-ketoglutarate; Asp, aspartate; CFU, colony-forming units; Gln, glutamine; Glu, glutamate; Gly, glycine; GSH, glutathione; myo-I, myo-inositol; n.s. not significant; PC, phosphocholine; ppm, parts per million; Suc, succinate; tCho, total choline.

To assess the sensitivity of Gln/Glu^Low^ and Gln/Glu^High^ GSCs to Gln deprivation, we cultured GSCs for 48 h in medium supplemented with or deprived of L-Gln. The representative ^1^H NMR spectra (Figure 2a) and relative quantifications (Figure 2b) of metabolic extracts showed severely reduced Gln levels upon starvation. Moreover, Glu levels were reduced (JHH520: 42%, 407: 40%, GBM1: 53%, RAV19: 55%) in starved GSCs. Gln starvation significantly reduced the abundance of the TCA cycle intermediate succinate (Suc) in all tested cell lines, and resulted in lower concentrations of aspartate (Asp), an amino acid which is synthetized in a Glu-dependent reaction. Contrarily, the oncometabolites phosphocholine (PC), total choline (tCho), myo-inositol (myo-I), and glycine (Gly) increased during starvation, suggesting that the GSCs switched to a more aggressive metabolic phenotype in order to cope with nutrient deprivation. To assess whether Gln starvation interferes with GSC maintenance, we measured in vitro clonogenicity and cell growth in Gln deprived and control conditions. Upon starvation, clonogenicity and cell growth were significantly reduced in Gln/Glu^Low^ GBM1 and RAV19 cells but not significantly affected in Gln/Glu^High^ GSCs (Figure 2c,d). In order to test whether starvation sensitivity is mediated by insufficient influx into the TCA cycle or is, at least in part, due to impaired Glu availability for the synthesis of Glu-dependent amino acids and antioxidants, we attempted to rescue the starvation phenotype of Gln/Glu^Low^ cells through the addition of alpha-ketoglutarate (αKG) or Glu. In GBM1 and RAV19 cells, the starvation sensitivity was reduced by 40 mM Glu and 40 mM αKG, whereas only Glu completely restored resistance in both cell lines (Figure 2e).

### 3.3. Overexpression of the Glutamate/Cystine Antiporter xCT in Gln/Glu^High^ GSCs Prevents Accumulation of Reactive Oxygen Species (ROS) during Starvation

**Figure 3:**
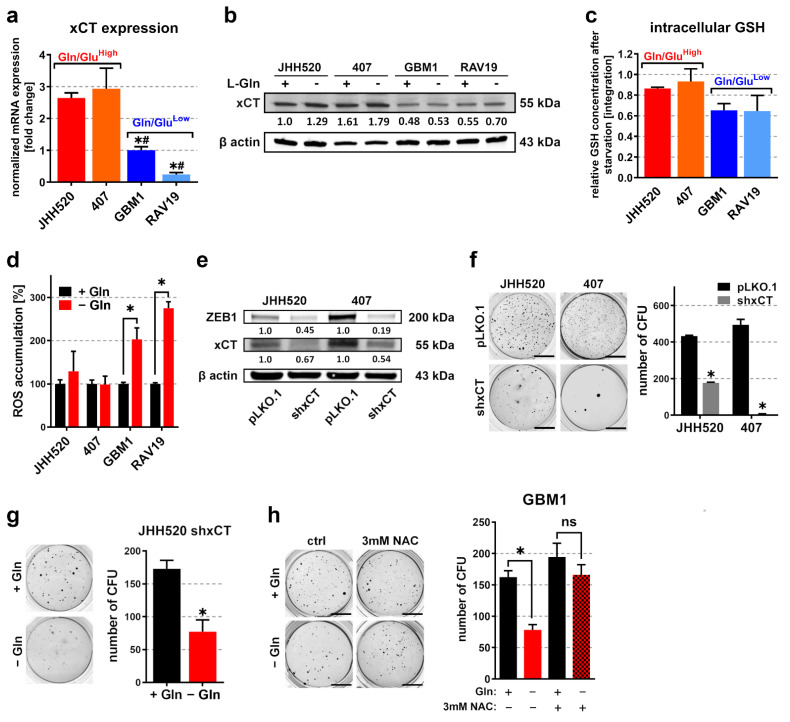
xCT-dependent ROS clearance mediates starvation resistance. Both mRNA (**a**) and protein (**b**) expression of the glutamate/cystine antiporter xCT were elevated in GSCs with high Gln/Glu ratios (JHH520 and 407) (loading control = β actin) (*n* = 3, significance was calculated with a one-way ANOVA, * significantly decreased (*p* < 0.05) compared to JHH520, # significantly decreased (*p* < 0.05) compared to 407). (**c**) Relative GSH concentrations of L-Gln-starved cells (compared to controls in full medium) assessed with ^1^H-NMR spectroscopy of metabolic extracts (*n* = 3, significance calculated with one-way ANOVA). (**d**) ROS accumulation in L-Gln-starved and control cells measured by means of DCF fluorescence intensity (*n* ≥ 3). (**e**) Immunoblotting verification of shRNA-based xCT knockdown and ZEB1 expression in shxCT and pLKO.1 (control) cells (loading control = β actin). (**f**) Soft agar colony formation in shxCT and pLKO.1 (control) cells (*n* = 3). (**g**) Soft agar colony formation in JHH shxCT cells cultured in the presence or absence of L-Gln (*n* = 3). (**h**) Soft agar colony formation in GBM1 cells starved for L-Gln in either the presence or absence of 3 mM NAC. For all soft agar assays, representative pictures of NBT-stained colonies and quantifications of three assays are shown (*n* = 3, scale bar equals 1 cm). For all assays, the data are presented as mean ± SD. Significance was calculated with a one-sided *t*-test if not specified elsewhere, * *p* < 0.05. Abbreviations: ctrl, control; CFU, colony-forming units; Gln, glutamine; GSH, glutathione; NAC, N-acetylcysteine; n.s., not significant; ROS, reactive oxygen species. Supplementary Figure 2 (S2) corresponds to Figure 3b, Supplementary Figure 3 (S3) corresponds to Figure 3e.

Considering that Glu rescued the starvation phenotype of Gln/Glu^Low^ GSCs more efficiently than αKG, we hypothesized a role for Glu in starvation sensitivity that may be independent of the TCA cycle. It is well known that Glu not only provides intermediates of the TCA cycle but also plays a crucial role in promoting reactive oxygen species (ROS) clearance. An important step in glutathione (GSH) synthesis is the export of Glu, which is mediated by the glutamate/cystine antiporter SLC7A11/xCT. Therefore, we next focused on xCT mRNA and protein evolution in the GSC lines. Interestingly, the starvation-resistant Gln/Glu^High^ JHH520 and 407 cells showed increased xCT mRNA and protein levels (Figure 3a,b). Moreover, starvation decreased intracellular GSH levels more efficiently in GBM1 (34%) and RAV19 (36%) GSCs compared to JHH520 (14%) and 407p (7%) GSCs (Figure 3c). To determine the possible association with ROS, we performed DCFDA-based ROS assays and observed significant ROS accumulation in sensitive (GBM1 and RAV19) but not in resistant (JHH520 and 407) GSCs (Figure 3d). To further analyze the relevance of xCT in starvation resistance, we performed shRNA-based knockdowns of xCT in high-expressing JHH520 and 407 cells and verified knockdown efficiency using Western blot analysis (Figure 3e). Interestingly, knockdown of xCT strongly reduced ZEB1 protein expression and the in vitro clonogenicity (Figure 3e,f). We further exposed JHH520 shxCT cells to glutamine starvation and measured the in vitro clonogenicity (Figure 3g). We observed a restoration of starvation sensitivity, since starved JHH520 shxCT cells exhibited significantly lower clonogenicity. Of note, the cell line 407 was excluded for similar analysis because it exhibited nearly no clonogenic capacity after knockdown of xCT. Subsequently, we starved sensitive GBM1 cells in the presence of the ROS inhibitor N-acetyl-L-cysteine (NAC) and observed no significant decrease in clonogenicity (Figure 3h). RAV19 cells responded with cell death to NAC-dependent medium acidification, so they were not considered for this particular assay. Overall, the data suggest that the sensitivity to Gln deprivation is due to the inadequate removal of ROS in GSCs with low xCT expression.

### 3.4. Differentiation of Highly Clonogenic Gln/Glu^High^ Cells Decreases xCT Expression and Restores Starvation Sensitivity

**Figure 4:**
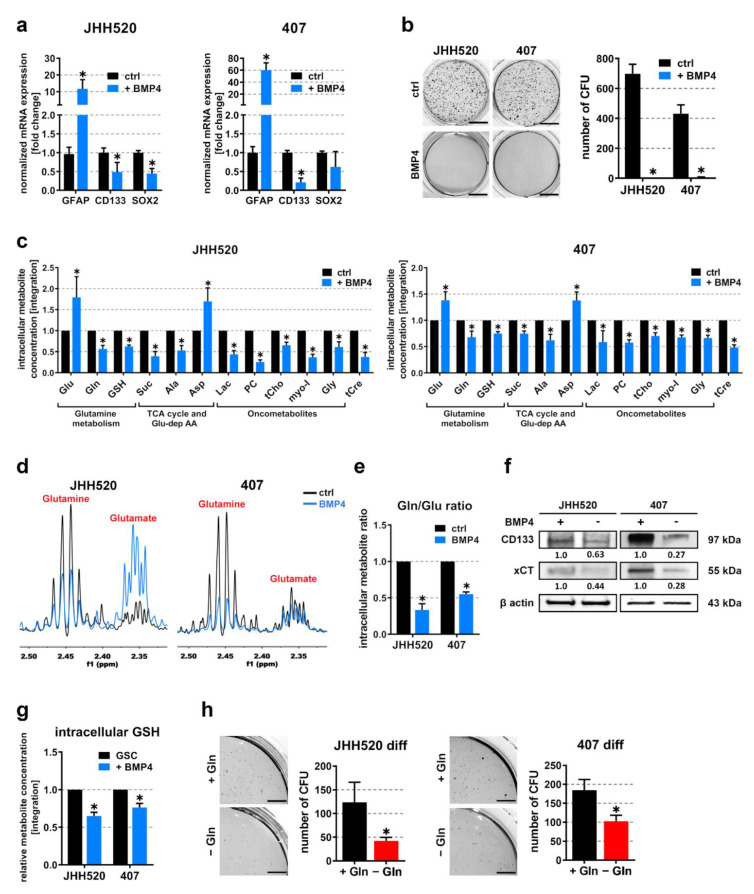
Differentiation of GSCs restores starvation sensitivity. (**a**) Differentiation of highly clonogenic GSC neurospheres (JHH520 and 407) was verified by means of quantitative real-time PCR, showing increased expression of GFAP and reduced expression of CD133 and SOX2 after 48 h treatment with 50 ng/mL BMP4 and (**b**) by soft agar colony formation assays, showing diminished in vitro clonogenicity upon 50 ng/mL BMP4 treatment (*n* = 3). Scale bar equals 1 cm. (**c**) Relative metabolite concentrations from metabolic extracts of GSC neurospheres cultured in the presence of solvent control (black) or 50 ng/mL BMP4 (blue) (*n* = 3). (**d**) Expanded regions from ^1^H-NMR spectra showing Gln and Glu multiplets and (**e**) corresponding Gln/Glu ratios of differentiated (BMP4) and control GSC cultures (*n* = 3). (**f**) Differentiation of GSCs with 50 ng/mL BMP4 for 48 h reduced CD133 and xCT protein levels, as assessed with immunoblotting (loading control = β actin). (**g**) Relative GSH concentrations in differentiated (BMP4) and control GSCs as assessed with ^1^H-NMR spectroscopy of metabolic extracts (*n* = 3). (**h**) Soft agar colony formation in differentiated (50 ng/mL BMP4) GSCs cultured in either the presence or absence of L-Gln. Due to the small colony size of differentiated GSCs, expanded regions of wells were chosen. Scale bar equals 0.5 cm. For all soft agar assays, representative pictures of NBT stained colonies and quantifications are shown (*n* = 3). For all assays, the data are presented as mean ± SD. Significance was calculated with Student’s *t*-tests, * *p* < 0.05. Abbreviations: AA, amino acids; Ala, alanine; Asp, aspartate; BMP4, bone morphogenic protein 4; CFU, colony forming units; ctrl, control; diff, differentiated; Gln, glutamine; Glu, glutamate; Gly, glycine; GSH, glutathione; Lac, lactate; myo-I, myo-inositol; n.s. not significant; PC, phosphocholine; ppm, parts per million; Suc, succinate; TCA, tricarboxylic acid; tCho, total choline; tCre, total creatine. Supplementary Figure 4 (S4) corresponds to Figure 4f.

Bone morphogenic protein 4 (BMP4) has previously been shown to effectively reduce the GSC pool in GBM [30]. As a proof of principle, we treated highly clonogenic JHH520 and 407 cells with 50 ng/mL BMP4 for 48 h and observed significantly increased expression of the differentiation marker glial fibrillary acidic protein (*GFAP*) and reduced expression of two stemness markers (*CD133* and *SOX2*). (Figure 4a). Importantly, we also noticed diminished clonogenic capacity in these cell lines upon BMP4 treatment (Figure 4b). Moreover, the relative metabolite concentrations of differentiated (BMP4) and control GSC cultures examined via ^1^H-NMR spectroscopy of metabolic extracts showed significantly reduced levels of the oncometabolites Lac, PC, tCho, Myo-I, and Gly (Figure 4c). Moreover, intracellular Suc and Ala levels were also impacted. As for glutamine metabolism, we observed an increase in Glu and a decrease in Gln and GSH levels, resulting in significantly decreased Gln/Glu ratios (Figure 4d,e). Immunoblotting experiments further revealed that differentiation with BMP4 not only reduced the expression of the GSC marker CD133 but also greatly decreased xCT levels (Figure 4f). Upon observing significantly reduced GSH levels in differentiated JHH520 and 407 cells (Figure 4g), we assumed that xCT mediates starvation resistance through efficient ROS clearance in highly clonogenic GSCs and speculated that differentiation would restore sensitivity to Gln starvation. As expected, we found that starvation of JHH520 and 407 cells in the presence of BMP4 restored their sensitivity to Gln deprivation, resulting in a significant decrease in clonogenicity (Figure 4h). Thus, it can be concluded that xCT is a marker for highly clonogenic GSCs, which are resistant to nutrient stress due to the efficient removal of ROS. Furthermore, our findings indicate a direct correlation between starvation resistance, xCT expression, and high Gln/Glu ratios in GSCs.

### 3.5. High Gln/Glu Ratios in Primary GBM Tumors Correlate with Increased Expression of xCT and ZEB1

**Figure 5:**
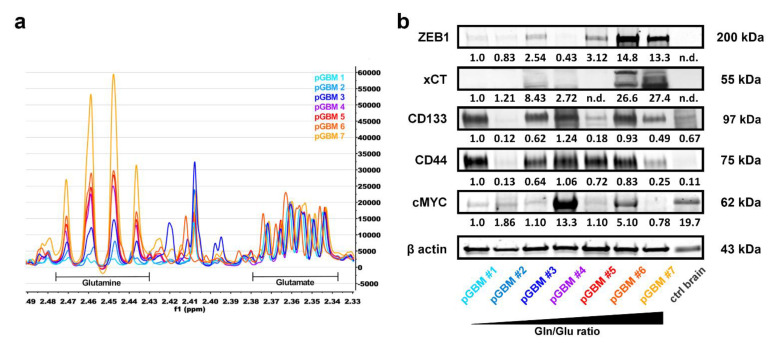
High Gln/Glu ratios predict ZEB1 and xCT expression in primary GBM tumors. (**a**) Expanded regions from ^1^H-NMR spectra showing Gln and Glu multiplets of metabolic extracts from seven primary glioblastoma (pGBM) patient samples. Spectra were normalized to the Glu content to visualize the differences in Gln/Glu ratios. (**b**) Immunoblotting of ZEB1, xCT, CD133, CD44, and cMYC protein in GBM patient samples arranged according to their Gln/Glu ratios in ascending order (loading control = β actin). A non-neoplastic brain sample of a trauma patient was blotted as a control. Abbreviations: ctrl, control; Gln, glutamine; Glu, glutamate; n.d., not detectable; pGBM, primary glioblastoma; ppm, parts per million. Supplementary Figures 5 (S5) and 6 (S6) correspond to Figure 5b.

Our aforementioned in vitro analyses revealed a close coherence between high intracellular Gln/Glu ratios and xCT-dependent ROS clearance in GSC cultures. To validate these results, we extended our analyses to primary GBM samples. Here again, we extracted water-soluble metabolites from seven primary GBMs and analyzed the samples with ^1^H-NMR spectroscopy. The analysis revealed substantial differences in Gln/Glu ratios between the specimens (Figure 5a). We did not observe any correlation between stem cell markers CD133, CD44, and the Gln/Glu ratio in the samples (Figure 5b). The primary tumors also showed a lack of correlation between c-Myc expression and high Gln/Glu ratios. However, similarly to GSC cultures, we found a strong association between high Gln/Glu ratios and increased expression of ZEB1 and xCT in primary GBM tumor tissue. Of interest, ZEB1 and xCT were significantly overexpressed in GBM samples, whereas they were absent in the control brain sample, further highlighting their importance in the cancer landscape.

## 4. Discussion

It has been nearly two decades since glioblastoma stem cells (GSCs) were first discussed in the literature [31,32], yet their distinct role in tumor heterogeneity and resistance to therapy continues to be of major concern. In particular, over the years, there has been considerable evidence that metabolic vulnerabilities play an important role in tumor progression and are associated with GSCs in yet-to-be-known pathways. However, the bioenergetic and biosynthetic requirements of GSCs are still unclear. In the current study, we sought to understand the metabolic dependencies of GSCs using high-resolution proton magnetic resonance (^1^H-NMR) spectroscopy. In a defined experimental framework, we stratified our in vitro GSC models into two subtypes (Gln/Glu^High^, Gln/Glu^Low^) primarily based on their relative amount of glutamine to glutamate (Gln/Glu). Subsequently, using diverse molecular approaches we performed a comprehensive analysis on four GSC neurosphere cultures (Gln/Glu^High^ JHH520 and 407; Gln/Glu^Low^ GBM1, and RAV19) and primary GBM samples.

We first showed that the in vitro clonogenicity of GSCs directly correlated with high Gln/Glu ratios and increased intracellular glutamine concentrations. Interestingly, the protein expression of ZEB1, a transcription factor that drives epithelial-mesenchymal transition (EMT) and is involved in increased invasiveness and GSC accumulation [29], was found to be associated with the two subtypes that we had defined. In particular, the high clonogenic Gln/Glu^High^ cells (JHH520 and 407) showed elevated levels of ZEB1, whereas the low clonogenic Gln/Glu^Low^ cells (GBM1 and RAV19) expressed significantly less ZEB1 protein. In addition, we observed increased expression of the oncogene c-Myc (a regulator of glucose and glutamine metabolism) in Gln/Glu^High^ GSCs. However, the expression of another putative GSC marker, prominin/CD133, showed no correlation with the in vitro clonogenicity of GSC cultures. This can partially be explained by the lower engagement of CD133 or the predominance of other markers in the cultured conditions. Importantly, compared to the prominent suggested GSC markers CD133, SOX2, and CD44, our study emphasizes that EMT and stemness marker ZEB1 is the most reproducible marker associated with functional stemness, thereby further promoting this transcription factor as a powerful target in neuro-oncology. Next, we cultured GSCs with or without L-Gln and observed that Gln starvation significantly reduced the abundance of the TCA cycle intermediate succinate (Suc) and resulted in lower concentrations of aspartate (Asp), an amino acid synthetized in a Glu-dependent reaction. On the contrary, oncometabolites phosphocholine (PC), total choline (tCho), myo-inositol (myo-I), and glycine (Gly) were found to be elevated during starvation, suggesting that the starved GSCs shift to a more aggressive metabolic phenotype in order to cope with nutrient deprivation. In our attempt to rescue the starvation phenotype of Gln/Glu^Low^ cells through the addition of the TCA cycle intermediate alpha-ketoglutarate (αKG) or Glu, we observed that Glu alone was able to fully restore resistance in starvation-sensitive GBM1 and RAV19 cells. In other words, since the starvation phenotype of Gln/Glu^Low^ GSCs is more efficiently rescued by Glu than by αKG, it is reasonable to assume that the starvation sensitivity is at least partly due to TCA cycle-independent fates of Glu.

In addition to its importance for bioenergetic and biosynthetic processes, Glu is essential for the maintenance of a balanced redox homeostasis. Therefore, we addressed the potential role of glutamate/cystine antiporter SLC7A11/xCT-mediated Glu export during GSH production, which is crucial for the clearance of ROS. The glutamate/cystine antiporter xCT exports Glu in exchange for cystine, thereby facilitating GSH synthesis. Emphasizing the importance of xCT for cellular homeostasis, according to the results of a previous study, it was estimated that up to half of the intracellular Glu is exported via this route [33]. Of interest, we found that the starvation-resistant Gln/Glu^High^ JHH520 and 407 cells showed increased xCT mRNA and protein levels. Consistently with previous findings [34,35,36], our results clearly demonstrate that xCT expression correlates with aggressiveness, chemoresistance, and stem cell features in GBM. Moreover, the essential role of xCT-mediated Glu export in mediating sensitivity to nutrient deficiency is not specific to GBM and was observed in other tumor entities as well [37,38,39,40]. Furthermore, significant ROS accumulation was observed in the sensitive (GBM1 and RAV19) but not in resistant (JHH520 and 407) GSCs. A possible elevated Glu export in starvation-resistant Gln/Glu^High^ GSCs could explain the metabolic composition and the efficient ROS clearance in this subtype, which stands in strong opposition to the features we observed in Gln/Glu^Low^ cells. Elevated Glu export could also be the reason for the higher Gln/Glu ratios in the GSC lines expressing higher xCT levels. Our results are further supported by previous findings indicating that ROS accumulation drastically reduces the GSC pool, whereas efficient ROS removal promotes stem cell formation [41,42,43], providing a potential explanation for the reduced clonogenicity we observed in Gln/Glu^Low^ GBM1 and RAV19 cells. Furthermore, we found that starved GBM1 cells did not exhibit a significant decrease in clonogenicity in the presence of the ROS inhibitor N-acetyl-L-cysteine (NAC). This is consistent with a previous study reporting that NAC can suppress GSC differentiation and reverse the anti-stemness effects of ROS-inducing agents [44]. The importance of glutamine metabolism in GBM aggressiveness is further supported by the previous findings of our group, where we observed that the expression of glutaminase (GLS), the enzyme converting Gln into Glu, correlated with the aggressiveness of GSCs and pharmacological GLS inhibition reduced the clonogenic potential [28]. Overall, our data suggest that the sensitivity to Gln deprivation is due to inadequate removal of ROS in GSCs with low xCT expression. These cells can be identified non-invasively by measuring the Gln/Glu ratios using magnetic resonance spectroscopy. As a proof of principle, we further induced the differentiation of GSCs using BMP4, which is known to effectively reduce the GSC pool in GBM. As a result, the expression of the mature astrocytic marker GFAP was significantly increased, whereas the expression of stem cell markers CD133, and SOX2, as well as the oncometabolite levels of lactate (Lac), PC, tCho, Myo-I, and Gly, were markedly reduced. Again, we could associate stemness with xCT, since BMP4-mediated differentiation reduced both GSC clonogenicity and xCT protein expression. Importantly, the observed correlation between the metabolic composition of GSCs (especially Gln/Glu ratios) and the in vitro clonogenicity indicates a direct link between glutamine metabolism, ROS maintenance, and stemness character in GBMs, which requires further attention in order to develop GSC-based therapies. At the molecular level, we recently reported that changes in oncometabolites involved in choline metabolism were associated with ZEB1 expression and in vitro clonogenicity [23]. Hence, the further characterization of the measured GSC-dependent oncometabolites is warranted to characterize GSCs in more detail and reveal dependencies that could be used in therapy development.

To validate our results from the GSC cell lines, we extended our analyses to primary GBM samples and first confirmed significant differences in Gln/Glu ratios between the samples. As with the results in GSC cultures, we did not observe any correlation between stem cell markers CD133, CD44, and the Gln/Glu ratio in the samples. Nevertheless, similarly to the GSC cultures, we found a strong association between high Gln/Glu ratios and increased expression of ZEB1 and xCT in primary GBM tumor tissues. Interestingly, ZEB1 and xCT were significantly overexpressed in GBM samples, whereas they were absent in the control brain sample, further highlighting their importance in the cancer landscape. The differences we observed in the primary GBM samples may indicate a correlation with the different GBM subtypes based on either immunohistochemical or molecular characterization [45,46]. However, a larger cohort with multiple subtypes (IDH1 mutation, MGMT methylation etc.) and the data from diverse classified GBM cell lines (classical, proneural, and mesenchymal) will be required to determine this.

## 5. Conclusions

Our analysis suggests that monitoring the Gln/Glu ratio and xCT expression in glioblastoma can predict the existence of highly aggressive GSCs and the tumor’s resistance to Gln starvation. In vivo validation is required to verify the relevance of this finding for diagnostic purposes. We also propose that minimally invasive, label-free metabolomics, together with genetic screening, can improve tumor subtype stratification to further support patient care.

## Data Availability

All data will be made available upon request by the reader.

## Supplementary Materials

The following are available online at https://www.mdpi.com/article/10.3390/cancers13236001/s1. The original Western blots can be seen in the supplementary files (S1–S6). Supplementary Figure 1 (S1) corresponds to Figure 1e, S2 corresponds to Figure 3b, S3 corresponds to Figure 3e, S4 corre-sponds to Figure 4f, and S5 and S6 correspond to Figure 5b in the main manuscript.
