# Supplementary figures and images for: Overexpression of Cystine/Glutamate Antiporter xCT Correlates with Nutrient Flexibility and ZEB1 Expression in Highly Clonogenic Glioblastoma Stem-like Cells (GSCs)
Source: Cancers (Basel). 2021 Nov 29;13(23):6001. doi: 10.3390/cancers13236001 (PMC8672273; doi:10.3390/cancers13236001)

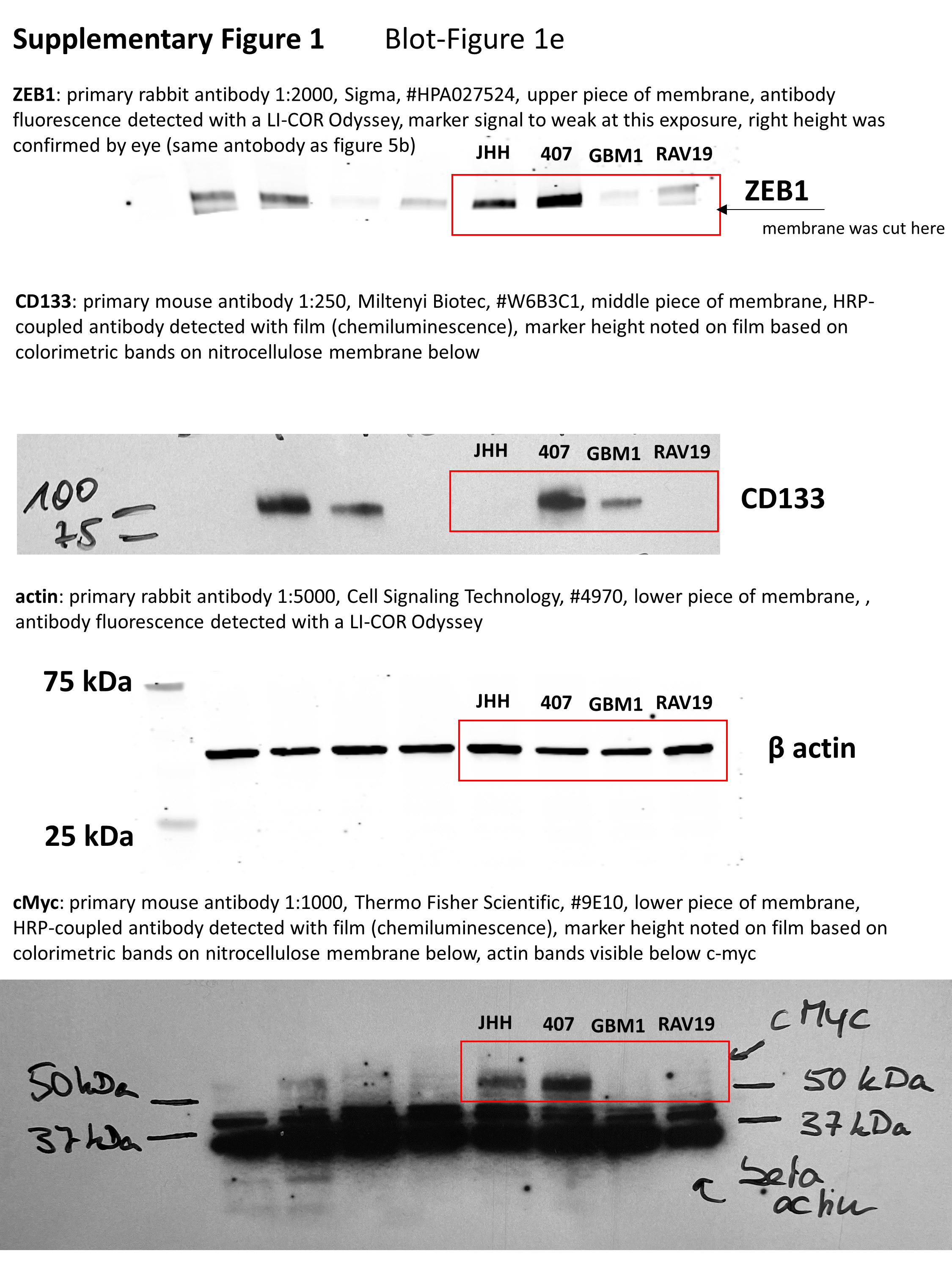

Supplement: Supplementary file 1 [file cancers-13-06001-s001.zip › Supplementary figure 1.TIF]

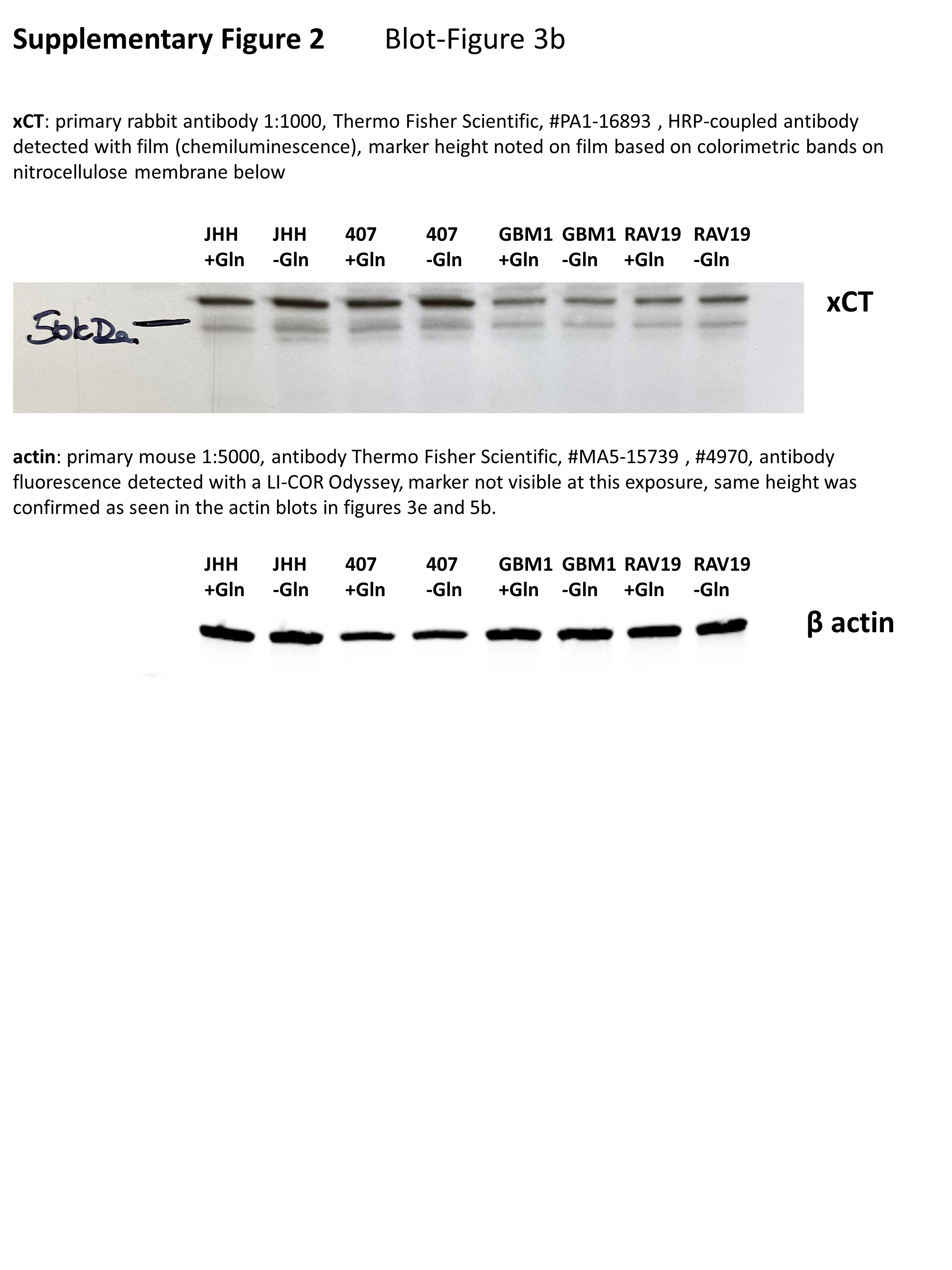

Supplement: Supplementary file 1 [file cancers-13-06001-s001.zip › Supplementary figure 2.TIF]

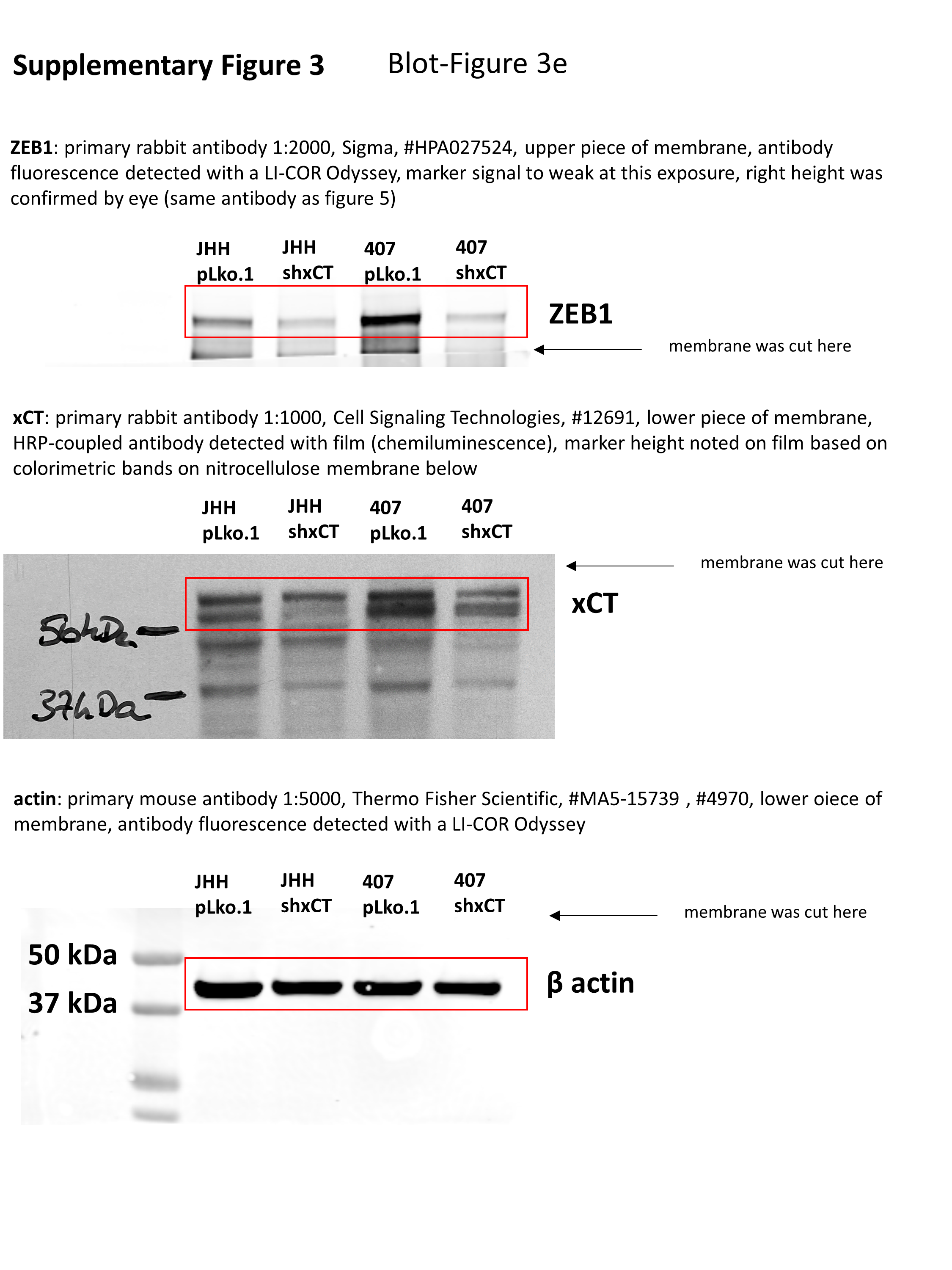

Supplement: Supplementary file 1 [file cancers-13-06001-s001.zip › Supplementary figure 3.TIF]

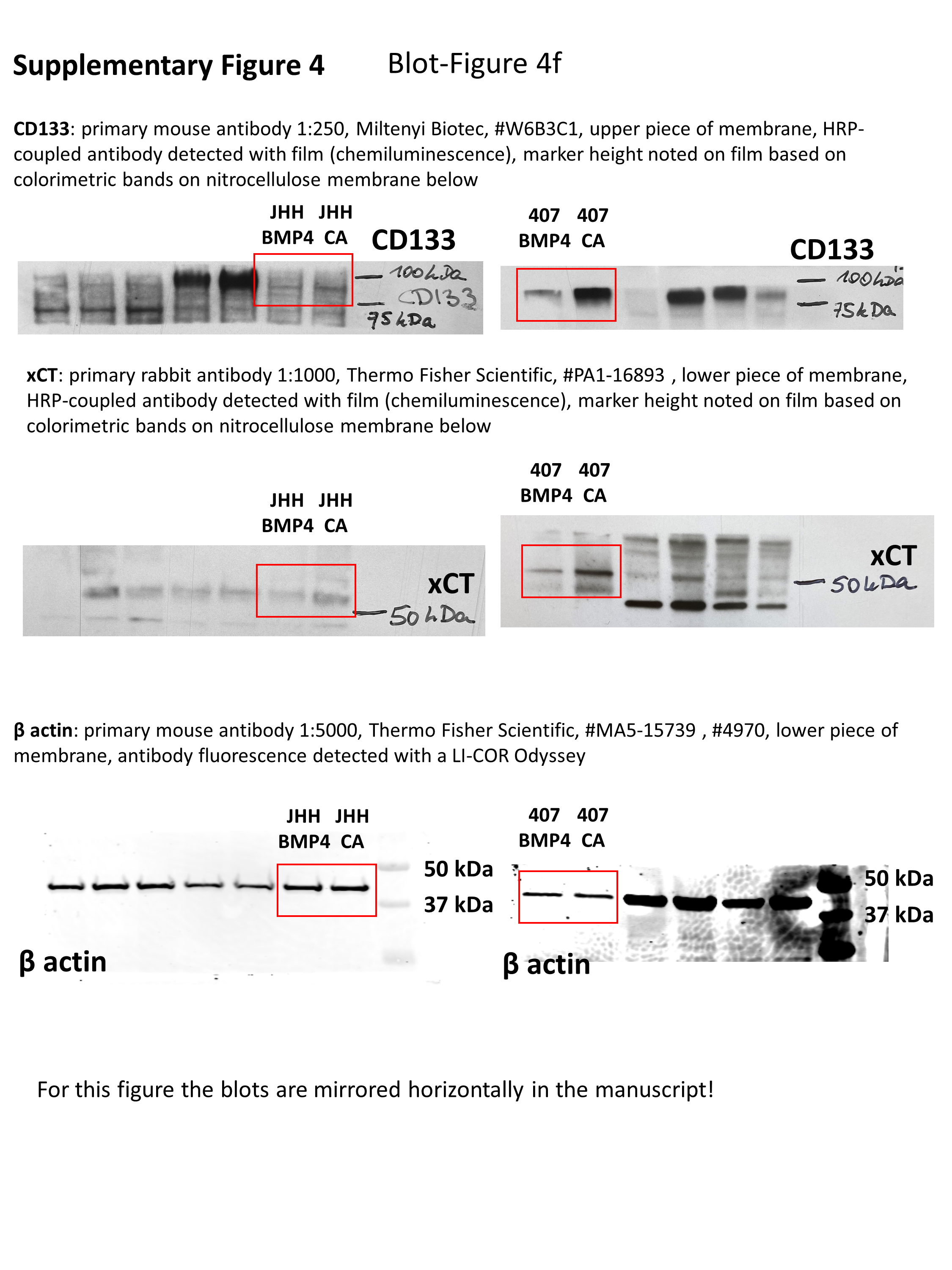

Supplement: Supplementary file 1 [file cancers-13-06001-s001.zip › Supplementary figure 4.TIF]

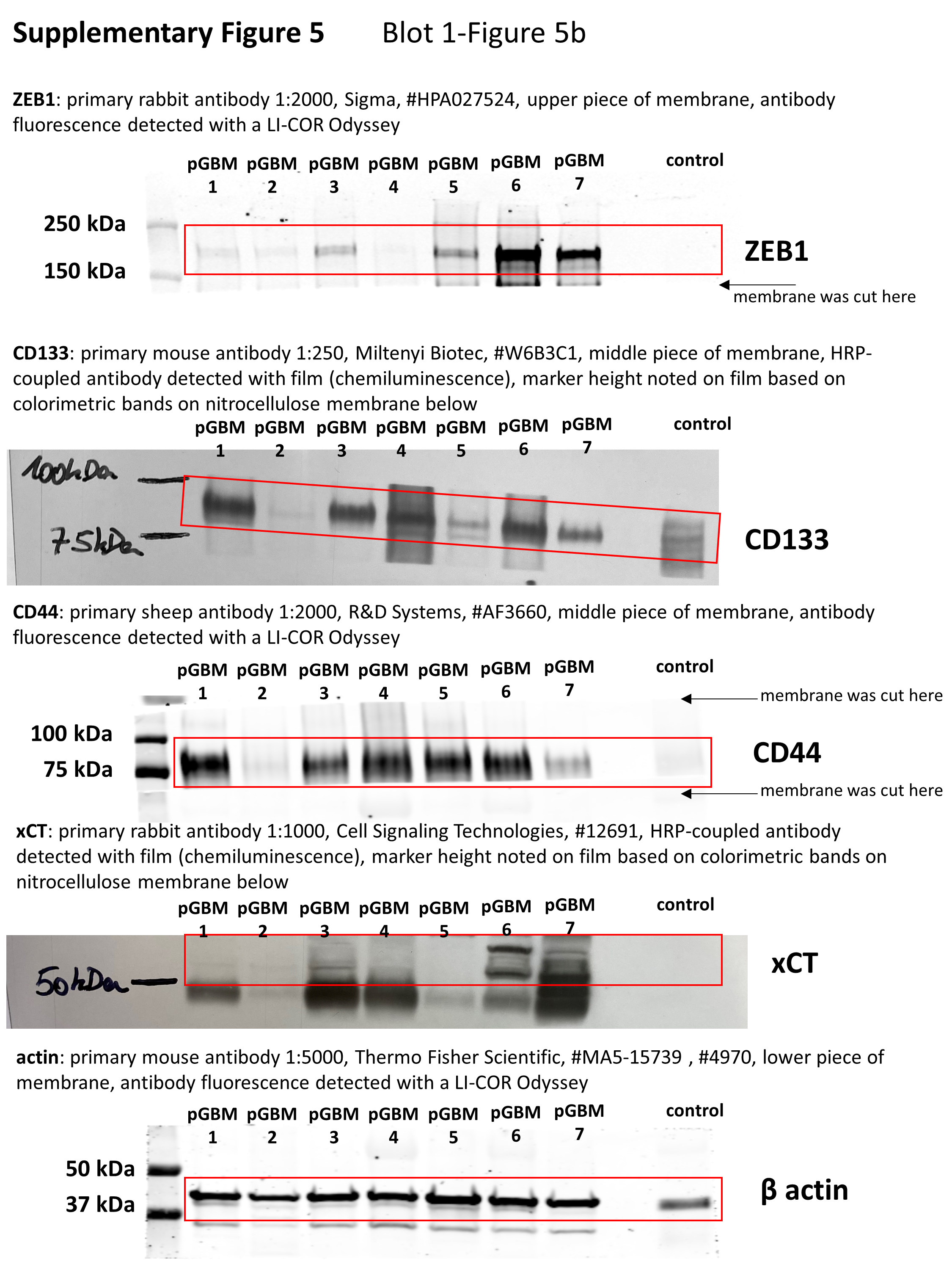

Supplement: Supplementary file 1 [file cancers-13-06001-s001.zip › Supplementary figure 5.TIF]

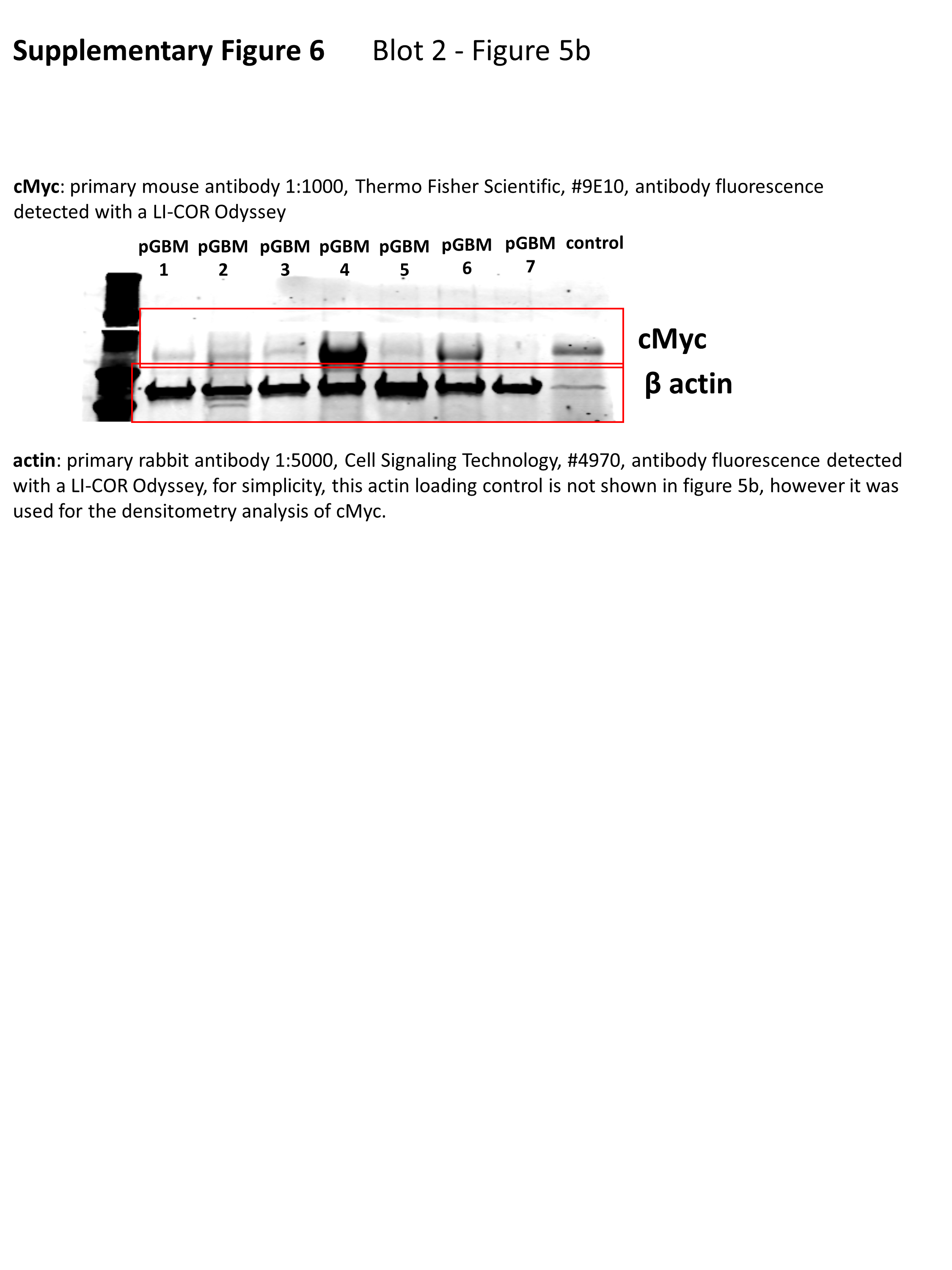

Supplement: Supplementary file 1 [file cancers-13-06001-s001.zip › Supplementary figure 6.TIF]
